# Hematospermia and Cloacogenic Transitional Cell Carcinoma: A Twist on Significance and Meaning

**DOI:** 10.1155/2016/8050459

**Published:** 2016-02-08

**Authors:** Alex M. Hennessey, Jessica M. Clement, Faripour Forouhar, John A. Taylor

**Affiliations:** ^1^School of Medicine, University of Connecticut Health Center, Farmington, CT, USA; ^2^Division of Hematology Oncology, University of Connecticut Health Center, Farmington, CT, USA; ^3^Department of Pathology, University of Connecticut Health Center, Farmington, CT, USA; ^4^Division of Urology, University of Connecticut Health Center, Farmington, CT, USA

## Abstract

A 52-year-old gentleman presented with recurrent hematospermia. Further history revealed recent onset of constipation and difficulty voiding. Rectal examination revealed a firm, polypoid mass and colonoscopy showed suspicious, ulcerated lesions of the rectal mucosa with narrowing of the rectal vault. Pathology demonstrated transitional cell carcinoma of the rectum. While transitional cell carcinoma is a common genitourinary cancer, its presence in the anus and rectum is exceedingly rare. Furthermore, hematospermia is generally not associated with malignancy. This case is a remarkable example of two pathologic processes presenting in rare form and underscores the value of a thorough investigation of hematospermia when associated with other clinical symptoms.

## 1. Introduction

Hematospermia, the presence of blood in the ejaculate, is generally considered benign. There are multiple possible etiologies ranging from inflammation and infection to structural abnormalities [1–7]. Only in about 2% of cases is hematospermia associated with malignancy of the genitourinary tract [1]. We present a patient initially referred for complaint of recurrent hematospermia who, on further evaluation, was found to have a transitional cell carcinoma of the rectum. While transitional cell carcinoma (TCC) is a common genitourinary cancer, its presence in the anus and rectum is rare, accounting for 2%-3% of anorectal cancers [8]. This case is a remarkable example of two pathologic processes presenting in rare form and underscores the value of a thorough investigation of hematospermia when associated with other clinical symptoms.

## 2. Case Report

**Figure 1:**
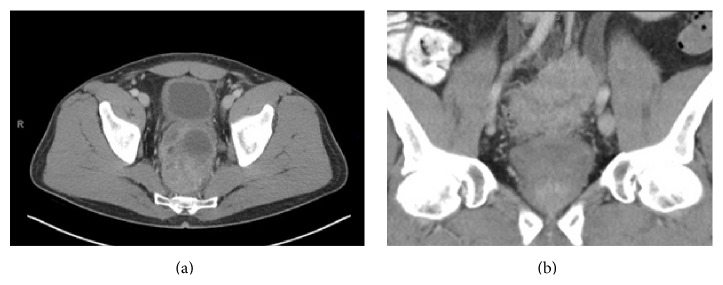
CT imaging of pelvis: (a) axial images showing large mass posterior to the bladder with areas of necrosis; (b) sagittal images with mass superior to bladder extending to involve the left ureter with proximal hydroureteronephrosis.

**Figure 2:**
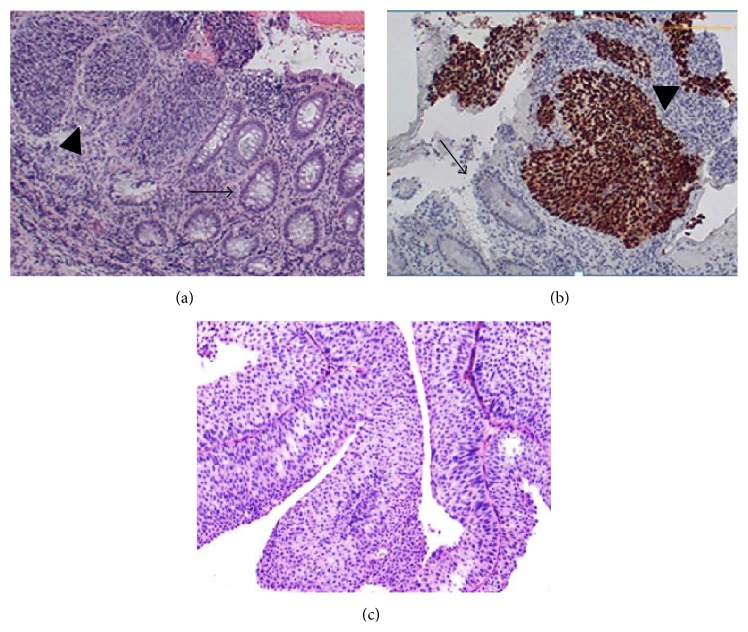
Representative images of (a) nests of flat, nonpapillary TCC (arrow head) within colonic epithelium (arrow) typical of cloacogenic TCC in rectum (H&E ×100). (b) p63 immunoperoxidase stain shows nuclear staining in the nests of cloacogenic TCC and negative staining in colonic epithelium (arrow) (×100). (c) High-grade papillary urothelial TCC (×100).

A 52-year-old man presented with a 6-week history of hematospermia, describing his ejaculate as brown in color. There was no history of trauma, and the patient denied any hematuria. On further questioning he reported a 3-month history of increasing difficulty with urination, which was often relieved after defecation. He had been concurrently experiencing constipation with decreased stool caliber. The patient had a past medical history of type II diabetes mellitus, fatty liver disease, arthritis, and seasonal allergies. He was a lifetime nonuser of tobacco products and reported rare recreational marijuana use. On rectal examination a firm, polypoid mass was palpated in the rectal vault with gross blood noted upon completion. The remainder of the examination was unremarkable. He was referred to gastroenterology for a colonoscopy and a CT scan was ordered. This demonstrated a large, lobular soft tissue mass in the lower central pelvis encasing the rectum and involving the posterior margin of the prostate (Figure 1(a)). The mass extended cephalad to involve the distal left ureter, resulting in moderate hydronephrosis (Figure 1(b)). There was left pelvic sidewall lymphadenopathy as well. Colonoscopy revealed edematous and erythematous folds causing rectal narrowing with small areas of ulceration and exudates, despite having a normal colonoscopy two years prior. Multiple biopsies were taken at the anorectal junction and pathology was consistent with high-grade TCC of the colonic epithelium (Figure 2). There was question of primary colonic TCC versus primary bladder carcinoma with secondary colonic involvement; however cystoscopy revealed normal anatomy and no evidence of primary bladder neoplasm.

With the most likely diagnosis of transitional cell cloacogenic carcinoma of the anorectal verge, the patient underwent treatment with gemcitabine and cisplatin administered every 21 days, similar to the traditional treatment for TCC of the bladder [9–12]. Restaging studies showed tumor regression and no development of metastatic disease. He is currently receiving 5-fluorouracil and mitomycin concomitant with radiation therapy given effectiveness in TCC [13].

## 3. Discussion

Hematospermia, while worrisome and distressing to patients, almost always represents a benign condition. The average age at presentation is 37, and it is seen in approximately 1 : 5,000 new patients presenting to a urology clinic [2]. However, it is difficult to truly assess the prevalence due to underreporting among patients.

Inflammation or infection accounts for 39%–55% of cases [3]. Only in very rare cases has hematospermia been seen in conjunction with malignancy. In most instances hematospermia is iatrogenic. The most common etiology is prostate biopsy, with hematospermia seen in 50–80% of men undergoing this procedure [4, 5]. Outside of biopsy, iatrogenic causes include external beam radiation therapy, brachytherapy, and vasectomy [7]. Inflammatory processes associated with hematospermia include perineal or pelvic trauma (i.e., repetitive injury such as cycling or chronic constipation) and calculi of the seminal vesicles, prostate, urethra, bladder, and ureters. Prolonged sexual abstinence has also been associated with hematospermia, as the expulsion of semen from a distended seminal vesicle can lead to mucosal irritation and bleeding [3]. Conversely, vigorous sexual intercourse or masturbation can lead to genital congestion and bleeding [6]. Finally, infections leading to prostatitis, urethritis, orchitis, epididymitis, and spermatocystitis also induce mucosal inflammation, leading to hematospermia. These cases are often associated with irritative genitourinary symptoms or pain [6].

Hematospermia has also been associated with systemic illnesses, such as hypertension, coagulation disorders, scurvy, amyloidosis, and cirrhosis. Vascular disorders such as prostatic varices or telangiectasias, urethral hemangioma, and AV malformations can also lead to hematospermia [6, 7]. Structural abnormalities leading to hematospermia include prostatic, seminal vesicle and ejaculatory duct cysts, ejaculatory duct obstruction, and urethral stricture.

Evidence of association with malignancy is scarce and often limited to case reports or case series. Such cases have included primary seminal vesicle adenocarcinoma [14], metastatic melanoma to the seminal vesicle [15], primary testicular cancer [16], and primary prostate cancer [17]. One retrospective analysis of 26,126 men of ages 50 and older demonstrated 19 cases of cancer in the 139 men who presented with hematospermia (13.7% incidence) versus 6.5% of cancer cases seen in men without hematospermia [18].

However, one literature review found only 33 tumors, 25 of them prostatic, among 931 cases of hematospermia—an incidence of 3.5% [7]. Similarly, a case series of 300 men with hematospermia showed only a 5.7% incidence of prostate cancer during long-term follow-up [19]. Virtually all cases of hematospermia associated with cancer are seen in men older than age of 40 years [6]. This is the first reported case of hematospermia as the presenting manifestation of anorectal carcinoma.

As most cases tend to be benign in origin, diagnostic workup is often minimal and catered to the individual patient. Some physicians recommend more thorough evaluation for hematospermia in men over age of 40, as there is higher likelihood of detecting a more serious disease entity [7]. Blood pressure should be evaluated. Thorough palpation and examination of penis, testes, epididymides, vasa deferentia, and urethra should be undertaken to evaluate for any structural abnormalities or suspicious lesions. Rectal examination allows for evaluation of the prostate—tenderness may suggest infection or inflammation, while nodularity or firmness could indicate a neoplasm. Concern for neoplasm may also warrant prostate specific antigen (PSA) testing as well as radiographic imaging. Transrectal ultrasonography (TRUS) can also allow for visualization of calculi, cysts, mullerian duct remnants, prostatic varices, and inflammatory changes. This may be a particularly useful imaging modality in men with chronic hematospermia [20, 21]. Urinalysis can rule out hematuria or infection.

Management is typically expectant, as the majority of cases will self-resolve. Underlying causes should be treated appropriately. Infections can be managed with appropriate antimicrobials, cysts of the prostate or seminal vesicle can be aspirated under TRUS guidance, and ductal obstructions may be resected transurethrally [6, 22].

TCC is a malignancy most commonly seen in the urogenital tract, where it is termed urothelial carcinoma. However, some cases of anorectal cancer are also of transitional cell origin and in this context are often referred to as “cloacogenic” or “basaloid” carcinoma. These rare tumors account for 2%-3% of all anorectal carcinomas and occur 1.5–2 times as often in females. The average patient age is 60 [8, 23, 24]. Some suggested etiologies include Crohn's disease, anal intercourse, and chronic fistula-in-ano. However, the patient presented in this case had no predisposing factors. Presenting symptoms are similar to those of other anorectal neoplasms and may include hematochezia, rectal pain, change in bowel habits, or anal mass [25].

The onset of symptoms is often abrupt, with the duration of symptoms for cloacogenic carcinoma being generally shorter in comparison to those for squamous cell anal neoplasms [25]. The patient described in this report had a normal colonoscopy two years prior to onset of symptoms. One study showed lymph node metastases at time of surgery in one-third of cases, and extranodal metastases in 19% of cases [24].

Cloacogenic carcinomas typically originate around the pectinate line of the anal canal, where the simple columnar epithelium of the upper third meets the stratified squamous epithelium of the lower two-thirds. At this junction, the columnar epithelium becomes cuboidal and gradually changes into transitional epithelium, extending to cover the anal valves just proximal to the pectinate line. The transitional epithelium of the urogenital aspect of the cloaca can be retained in the anal glands at the border of the pectinate line, thus explaining the presence of transitional epithelium in the anal canal [8, 26].

Anorectal TCC is sometimes considered a subset of anorectal squamous cell carcinoma (SCC) [27]. Due to its rarity, there is little research devoted specifically to the management of anorectal TCC. As such, treatment modalities have typically been based upon the Nigro protocol, which has been a long-held gold standard for anal SCC. This consists of 5-fluorouracil (5-FU), mitomycin (MMC), and radiation therapy [27, 28]. This protocol has also been demonstrated to improve control and survival rates of bladder TCC [13]. One small study of anal cloacogenic carcinoma found that 5-FU plus MMC or cisplatin led to three- and five-year survival rates of 71% and 48%, respectively [25]. Other studies have considered the addition of cisplatin to this regimen, with and without radiation, although they failed to show any superiority to the Nigro protocol for treatment of anal SCC [29]. Systemic gemcitabine in combination with cisplatin is considered a standard first-line regimen for patients with advanced TCC of the bladder [9–11].

## 4. Conclusions

This case is the first description of hematospermia due to invasive anorectal carcinoma and is instructive for the clinical assessment of patients presenting with hematospermia, although rare, regional, invasive disease originating outside the urinary tract may cause unusual presentations. It also reminds the clinician that TCC does not always arise from the urothelium and may manifest from the anorectal verge. As hematospermia is often considered benign and self-resolving, this case underscores the importance of investigation and workup when associated with other clinical symptoms.
